# Pharmacokinetic Comparability of a Biosimilar Trastuzumab Anticipated from Its Physicochemical and Biological Characterization

**DOI:** 10.1155/2015/874916

**Published:** 2015-11-19

**Authors:** Mariana P. Miranda-Hernández, Carlos A. López-Morales, Nelly Piña-Lara, Francisco C. Perdomo-Abúndez, Néstor O. Pérez, Jorge Revilla-Beltri, Aarón Molina-Pérez, Larisa Estrada-Marín, Luis F. Flores-Ortiz, Alejandro Ruiz-Argüelles, Emilio Medina-Rivero

**Affiliations:** ^1^Unidad de Investigación y Desarrollo, Probiomed S.A. de C.V. Cruce de carreteras Acatzingo-Zumpahuacán, 52400 Tenancingo, MEX, Mexico; ^2^Dirección Médica, Probiomed S.A. de C.V., Avenue Ejército Nacional No. 499, Colonia Granada, Delegación Miguel Hidalgo, 11520 México, D.F., Mexico; ^3^Laboratorios Clínicos, Puebla de Bioequivalencia, S.A. de C.V., Boulevard Díaz Ordaz No. 808, 72530 Puebla, PUE, Mexico

## Abstract

Comparability between a biosimilar and its reference product requires the evaluation of critical quality attributes that may impact on its pharmacological response. Herein we present a physicochemical characterization of a biosimilar trastuzumab focused on the attributes related to the pharmacokinetic response. Capillary isoelectrofocusing (cIEF) and cation exchange chromatography (CEX) were used to evaluate charge heterogeneity; glycosylation profiles were assessed through hydrophilic interaction liquid chromatography (HILIC); aggregates content was evaluated through size exclusion chromatography (SEC) while binding affinity to FcRn was evaluated using isothermal titration calorimetry (ITC). The biosimilar trastuzumab and its reference product exhibited a high degree of similarity for the evaluated attributes. In regard to the pharmacokinetic parameters, randomized, double blind, and two-arm parallel and prospective study was employed after the administration of a single intravenous dose in healthy volunteers. No significant differences were found between the pharmacokinetic profiles of both products. Our results confirm that similarity of the critical quality attributes between a biosimilar product, obtained from a different manufacturing process, and the reference product resulted in comparable pharmacokinetic profiles, diminishing the uncertainty related to the biosimilar's safety and efficacy.

## 1. Introduction

Recombinant biotherapeutic proteins, such as monoclonal antibodies (mAbs), require the establishment of an appropriate three-dimensional structure that allows the exposure of specific domains for the recognition of target sequences. This structure and its maintenance are dependent upon the protein chemical composition and the environments to which it is exposed [1].

During their biosynthesis, recombinant proteins are subject to posttranslational modifications that provide an inherent amount of heterogeneity in their molecular structure in addition to further modifications that can be acquired during the purification, formulation, and shelf life [2].

Although intrinsic batch-to-batch heterogeneity is present in mAbs [3], it can be controlled during their manufacturing process, allowing the establishment of acceptance ranges strongly associated with the pharmacological response of the drug, in order to have consistency and ensure the mAb's safety and efficacy.

Due to the continuous improvements in the biotechnological industry processes, several guidelines have been published to ensure that potential modifications associated with those developments, both within and among manufactures, do not have or potentially alter the pharmacological behavior or induce adverse effects on patients [4–6]. Furthermore, advances in physicochemical and functional characterization have increased the knowledge of protein's attributes and their relationship with pharmacological responses [7, 8]. Thereby, the assessment of physicochemical comparability among batches coming from process changes throughout a product-licensed life cycle [9] provides sufficient evidence to maintain its denomination and product label without the need of additional clinical studies [3]. This approach can be employed to demonstrate comparability of biosimilars and demarcate the extent of additional clinical assessment.

Trastuzumab is a humanized immunoglobulin isotype 1 (IgG1) targeted against the extracellular portion of the human epidermal growth factor receptor 2 (HER2, p185) which is overexpressed in approximately 15 to 30% of invasive breast cancer cases [10, 11]. This mAb contains murine complementary-determining regions (CDR) anti-p185 and human framework regions and as other recombinant proteins is susceptible to undergoing posttranslational modifications.

The primary sequence of trastuzumab contains 1328 amino acids with an averaged molecular mass of 148.3 kDa, one N-glycosylation site at Asn300 in a conserved sequence of the crystallisable fragment (Fc), occupied by biantennary glycan structures. The isoelectric point (pI) of its charge variants ranges from 8.0 to 8.9, whereas the main isoform pI is 8.7. This charge heterogeneity comes from the cyclization of the N-terminal glutamic acid of the heavy chain with the consequent formation of pyroglutamic acid, as well as from the oxidation of methionine residues, the deamidation/isomerization of asparagine and aspartic residues, C-terminal lysines, and microheterogeneity of glycan structures [12].

The aim of this study was to demonstrate that comparability of the pharmacokinetic behavior between a biosimilar and its reference product can be anticipated from their physicochemical and biological comparability. Thus, the comparability exercise between the biosimilar trastuzumab and its reference product focused on the physicochemical and functional attributes that have been reported to have an impact on the pharmacokinetic profile (PK) of monoclonal antibodies [8] such as charge and glycosylation heterogeneity, aggregates content, and binding affinity to FcRn. Subsequently, the exercise was complemented with a narrowed pharmacokinetics study conducted in healthy volunteers to confirm that comparability of the evaluated critical attributes is reflected on comparable pharmacokinetic behavior of the biosimilar trastuzumab and the reference product.

## 2. Materials and Methods

### 2.1. Materials

Acetonitrile (C_2_H_3_N), dibasic sodium phosphate heptahydrate (Na_2_HPO_4_·7H_2_O), monobasic sodium phosphate monohydrate (NaH_2_PO_4_·H_2_O), sodium chloride (NaCl), sodium azide (NaN_3_), TRIS-hydrochloride (NH_2_C(C_2_OH)_3_·HCl), phosphoric acid (H_3_PO_4_), and sodium hydroxide (NaOH) were obtained from J.T. Baker (Center Valley, PA). Ammonium formate (CH_5_NO_2_) and formic acid were obtained from Sigma-Aldrich (Saint Louis, MO). 2-Aminobenzamide (2-AB) was obtained from Prozyme Inc. (Hayward, CA) and PNGase F was purchased from New England Biolabs (Woburn, MA). Water was obtained from a Millipore Milli-Q Biocel system (Billerica, MA). All solutions were filtered through 0.2 *μ*m prior to analysis. Two brands of trastuzumab (440 mg powder for concentrate for solution for infusion) were employed: biosimilar Trastuzumab-Probiomed (Probiomed S.A. de C.V., Mexico) and Herceptin (F. Hoffmann-La Roche Ltd., Basel, Switzerland).

### 2.2. Methods

#### 2.2.1. Charge Variants Analyses

Charge variants analyses by cation exchange ultraperformance liquid chromatography (CEX-UPLC) and capillary isoelectric focusing (cIEF) were conducted as previously described by Espinosa-de la Garza et al. [13] and Flores-Ortiz et al. [14].

#### 2.2.2. Glycosylation Profile

Glycan release and derivatization were performed according to Mittermayr et al. [15]. Chromatographic separation was carried out using ACQUITY UPLC H-Class Bio System with a linear gradient from 22 to 50% acetonitrile using 100 mM ammonium formate solution at pH 4.50 (mobile phase A). Fluorescence detection was set at 250 nm and 420 nm excitation wavelength using 150 × 2.1 mm, 1.7 *μ*m ACQUITY UPLC BEH glycan column coupled to 1.7 *μ*m VanGuard BEH glycan precolumn from Waters Corp. (Milford, MA).

#### 2.2.3. Affinity Constants by Isothermal Titration Calorimetry (ITC)

Affinity constants under equilibrium (*K*_*a*_) were obtained using Nano ITC instrument from TA Instruments Inc. (New Castle, DE). 300 *μ*L of FcRn solution at 50 *μ*M was titrated with repeated injections of 1.9 *μ*L trastuzumab solution at 40 *μ*M until saturation at 25°C. NanoAnalyze Software v2.4.1 (TA Instruments Inc.) was used for the integration of heat signals and nonlinear regression analysis of the data.

#### 2.2.4. Aggregates by Size Exclusion Chromatography (SEC)

Separation was carried out on 4.6 mm × 300 mm ACQUITY Ethylene Bridged Hybrid 200 analytical column with particle and pore diameters of 1.7 *μ*m and 200 Å, respectively (Waters Corp., Milford, MA). 20 mM phosphate, 150 mM NaCl, and 3 mM NaN_3_ solution at pH 6.8 were used as mobile phase. UV detector was set at 280 nm in ACQUITY UPLC H-Class Bio instrument.

#### 2.2.5. Ethical Considerations for the Pharmacokinetics Study

The study was conducted in accordance with the regulations and ethical principles based on the Declaration of Helsinki [16]. The protocol for this study was registered in the Mexican National Registry of Clinical Studies (RNEC), México, D.F., number 133300410B0226. The protocol and the written informed consent were reviewed and approved by the Institutional Review Board/Independent Ethics Committee (IRB/IEC) (The National Bioethics Commission CONBIOÉTICA, Mexico, D.F., number 21CEI00120130605, the Federal Commission for Protection Against Health Risks COFEPRIS, Mexico, D.F., and Laboratorios Clínicos de Puebla de Bioequivalencia, Puebla Mexico). All the volunteers were notified on the risks and scope of this research and signed the written informed consent as required by the IRB/IEC.

#### 2.2.6. Pharmacokinetics Study Design

A randomized, double blind, and two-arm parallel and prospective study in healthy volunteers was designed in order to test pharmacokinetic comparability [8, 17] between the biosimilar trastuzumab and the reference product. The healthy volunteers were randomly assigned into two groups, one to test Trastuzumab-Probiomed and one to test Herceptin. A single dose of 2 mg/kg of trastuzumab was administered to each volunteer as intravenous infusion during 90 minutes. The study was conducted at Laboratorios Clínicos de Puebla de Bioequivalencia, S.A. de C.V., according to the Guideline for Good Clinical Practice of the International Conference on Harmonization [18].

#### 2.2.7. Exclusion and Inclusion Criteria

According to the dosage information and published reports of the reference product, the administration of trastuzumab to women of reproductive potential is not recommended [10, 19, 20]. For this study, 28 healthy male volunteers aged between 18 and 55 years were recruited. The selection criteria included no history of hypersensitivity to trastuzumab or previous therapy with murine antibodies; additionally it was required that their clinical history and physical examination, vital signs, chest X-rays, electrocardiogram, and routine laboratory tests (haematology and biochemistry) revealed no evidence of disease. Elimination criteria included nonattendance to two consecutive samples, adverse reactions to the drug, dietary or drug transgression, and development of illness during the study or voluntary retirement. Three subjects voluntarily dropped out from the study; hence it was completed with 25 volunteers.

#### 2.2.8. Sampling

Blood samples were drawn from the volunteers in vacuum tubes without anticoagulant at 0 h, 1.5 h, 5.5 h, and 192 h (8 days), 408 h (17 days), 600 h (25 days), 792 h (33 days), 984 h (41 days), 1200 h (50 days), and 1344 h (56 days) after the infusion. Trastuzumab has a half-life (*t*_1/2_) of approximately 28 days [21–23]; therefore, this sampling scheme covered twice the *t*_1/2_ of trastuzumab. The samples were centrifuged to obtain serum, which was transferred to cryovials that were stored in liquid nitrogen until the analyses were performed.

#### 2.2.9. Serum Detection of Trastuzumab

Trastuzumab concentrations were determined using Herceptin Trastuzumab/Anti-HER2 Humanized IgG ELISA Kit from Alpha Diagnostics International (San Antonio, TX), using a TECAN Freedom Evolyzer liquid handler instrument (Männedorf, Switzerland). Quantitation range of trastuzumab was from 0.5 ng/mL to 10 ng/mL. Before measurement, samples were diluted accordingly.

#### 2.2.10. Determination of Pharmacokinetic Parameters and Statistical Analyses

Mean trastuzumab serum concentrations were plotted to compare the serum levels throughout time between the biosimilar trastuzumab and its reference product. Maximum serum concentration (*C*_max_), mean residence time (MRT), and the area under the curve from time zero to infinite (AUC_0–*∞*_) were estimated through a noncompartmental approach using the WinNonlin software V 6.3 (Pharsight Corporation, Mountain View, CA). The AUC values were calculated using the trapezoidal model, while *C*_max_ values were experimentally determined. Deviations from normality were assessed through D'Agostino-Pearson test, which rejects normality if the significance level *α* ≥ 0.05. Similarity between pharmacokinetic profiles was assessed by a two-tailed Wilcoxon-Mann-Whitney *U* test as a nonparametric method for non-Gaussian data to test the null hypothesis (*H*_0_) of no significant differences among values; this test rejects *H*_0_ if the significance level *α* < 0.05.

## 3. Results and Discussion

### 3.1. Physicochemical Analyses

Pharmacological mechanisms of biotherapeutic proteins such as mAbs can be correlated to specific physicochemical and functional attributes that can be evaluated using an appropriate analytical platform [24]. Regarding pharmacokinetics, charge heterogeneity is one of the main attributes to be evaluated because it plays an important role on the recognition of binding sites and target receptors [12] through affecting electrostatic and hydrophobic interactions with charged groups of cells and tissues.

**Figure 1:**
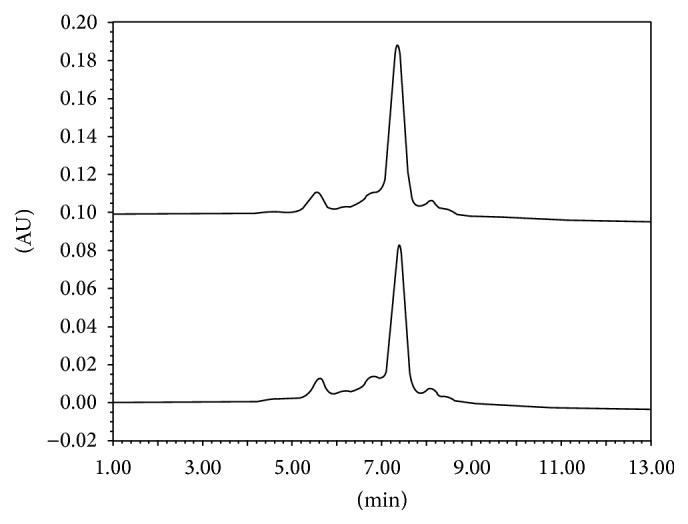
Cation exchange chromatograms of Trastuzumab-Probiomed (down) and its reference product (up).

Analyses of charge heterogeneity by CEX-UPLC showed correspondence of variants between the two products under study, as can be observed in Figure 1. The mean abundance of the main, acidic, and basic isoforms, obtained from three batches of each product, was 56.99%, 33.20%, and 9.81% for Trastuzumab-Probiomed and 62.48%, 27.26%, and 10.25% for the reference product, respectively. The relative abundance of acidic isoforms, due mainly to oxidations, deamidations, and glycations within the mAb [12], affects its distribution and clearance when administrated intravenously (IV). On the other hand, the presence of Lys C-terminal and aminations are mainly responsible for forming basic isoforms in the mAbs that could affect their clearance and tissue uptake; however, existing endogenous carboxypeptidases in the human bloodstream are capable of removing Lys C-terminal from mAbs within the first two hours after administration [25, 26]; therefore, basic isoforms are not expected to alter the PK of trastuzumab whose *t*_1/2_ is approximately 28 days [21–23].

A complementary approach to evaluate the relative abundance of charge variants can be achieved through an orthogonal technique such as cIEF for the analysis of the isoelectric point (pI). The pI for the main isoform was 8.69 ± 0.00 for Trastuzumab-Probiomed and 8.70 ± 0.01 for the reference product (Table 1) which is consistent with the expected pI variations, no larger than 0.2 units, to establish the identity of mAb. The global pI (i.e., average weighted from the relative abundance of pI isoforms) that could reveal major differences between manufacturing processes was 8.60 ± 0.01 for Trastuzumab-Probiomed and 8.64 ± 0.05 for the reference product (Table 1), confirming their similarity. It has been reported that global pI variations larger than 1 unit may impact on the PK and tissue distribution of the therapeutic protein [12, 27]. According to these results, pI variations between Trastuzumab-Probiomed and its reference product are not likely to show differences in their pharmacological responses.

Glycosylation is another attribute that could alter PK profiles that should be evaluated. For instance, the presence of highly mannosylated and hybrid glycoforms could affect clearance, AUC, and *t*_1/2_, besides affinity to the receptors involved in the mAb effector functions, probably due to charge or steric hindrances [8]. Although the aforementioned glycoforms are reported to alter clearance in mAbs, sialylation is also known to alter PK profiles in other biotherapeutics such as erythropoietin [28]. Therefore, the control of the cell culture conditions during biosynthesis that uphold an appropriate glycosylation profile is critical to produce a protein with comparable glycan microheterogeneity with respect to the reference product.  Table 2 shows the percentages of relevant glycoforms for PK of Trastuzumab-Probiomed and Herceptin. Both products exhibit comparable glycoform contents that are not expected to produce differences in their pharmacological behaviour. Additionally, afucosylated glycoforms, mostly relevant for pharmacodynamics profile, were also comparable, ranging from 8 to 13% for the reference product and from 10 to 15% for Trastuzumab-Probiomed.

The observed physicochemical comparability of Trastuzumab-Probiomed and the reference product suggest that their PK behaviour is expected to be the same.

Another relevant factor contributing to the clearance of a mAb is its interaction with the neonatal receptor (FcRn), which extends mAb half-life in serum by increasing IgG recycling to the circulation following endocytosis by endothelial cells through the binding to the Fc region. The binding affinity could be affected by chemical or structural modifications that alter the global mAb properties rather than those only located on the Fc region. The electrical dipole moment and the exposure of hydrophobic patches are responsible for the maintenance of the functional domains under a specific environment [29–31]; hence their evaluation is an indirect approach to predict its *t*_1/2_.

Isothermal titration calorimetry (ITC) can be used to estimate thermodynamic parameters such as enthalpy and entropy of trastuzumab-FcRn binding, which are used to calculate the stoichiometry and the affinity constant (*K*_*a*_) under equilibrium in solution. ITC results showed that Trastuzumab-Probiomed and the reference product affinities against FcRn rely within the same order of magnitude, being 2.21 × 10^6^ M^−1^ and 1.24 × 10^6^ M^−1^, respectively. These values are the closest estimation of the properties of the protein-protein interaction, because proteins remain solubilized and unconstrained while titrated in their native state, unlike other techniques based on immobilization that change or restrict the affinity.

In addition to charge and glycosylation heterogeneity, aggregates may be caused by the irreversible denaturation of the mAb that occurs during its manufacturing process and shelf life; thus it is a property to be determined and controlled. These aggregates influence the pharmacological responses such as PK and PD and immunogenicity due to the different structure of the mAb and an increased immune response denoted by the formation of neutralizing or nonneutralizing anti-drug antibodies [8]. In this regard, Trastuzumab-Probiomed and the reference product both exhibited aggregate levels lower than 2.0% (Table 3), which is in accordance with pharmacopeial specifications [32]. This suggests little risk of differential aggregate-based immunogenicity between Herceptin and Trastuzumab-Probiomed and consequently no impact on the PK.

### 3.2. Pharmacokinetics

The clinical phase was conducted successfully according to the protocol and the regulatory requirements. No severe side effects attributable to trastuzumab were observed during the study.

**Figure 2:**
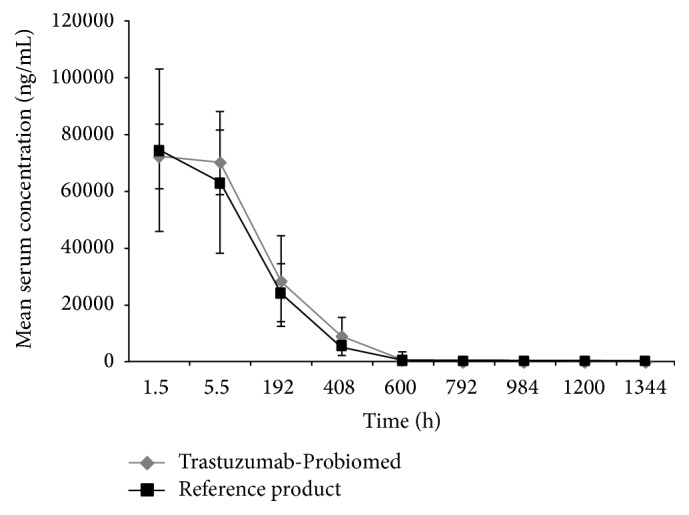
Mean serum concentrations of Trastuzumab-Probiomed and the reference product versus time. Measurements were obtained after the administration of an intravenous single dose of 2 mg/kg in healthy volunteers. Vertical bars depict standard deviations.

The suitability of the immunoassay employed for determining the serum concentrations of trastuzumab was proved by its selectivity towards IgGs against HER2, repeatability with RSD below 3.5%, and accuracy from 75 to 105% of recovery along the quantitation range (adjusted to a linear model, *r* ^2^ = 0.9843). Mean trastuzumab serum concentration versus time showed that both products followed the same trend from the time of administration and throughout the duration of the study (Figure 2).

Pharmacokinetics and pharmacodynamics of monoclonal antibodies are more complex compared to small-molecule drugs. For instance, mAbs elimination is generally dose dependent and mainly associated with FcRn transportation and antibody-antigen binding [33]. In addition, renal elimination is not relevant since IgG antibodies are eliminated via catabolism and endocytosis; in the specific case of trastuzumab it has been reported that its elimination is not affected in patients with renal failure [34].

The observed mean value of *C*_max_ was 74,428.3 ng/mL for the reference product, while for Trastuzumab-Probiomed it was 73,353.1 ng/mL. Mean MRT between the two products showed a difference of about 20 h; however this difference was within the variation range (SD = 49.2 h and 51.2 h) of both products. Mean AUC_0–*∞*_ for the reference product was 11,746,396.0 ng·h/mL, while for Trastuzumab-Probiomed it was 13,611,268.7 ng·h/mL. Results of the evaluated pharmacokinetic parameters are summarized in Table 4.

The D'Agostino-Pearson test indicated deviation from normality in the distribution of data; thus similarity between pharmacokinetic profiles by groups was achieved by the Wilcoxon-Mann-Whitney *U* test. Differences observed between the pharmacokinetic profiles among products were not significant as denoted by *p* values of the two-tailed *Z* scores for *C*_max_ and AUC_0–*∞*_ (Table 5).

The collective results from the physicochemical and functional characterization, along with the pharmacokinetics study, provided sufficient evidence to demonstrate that both products are comparable regarding their relevant attributes related to PK and that this comparability was reflected on comparable PK profiles. This demonstrates that the feasibility of the employment of a reliable platform comprised of state-of-the-art analytical techniques to assess the comparability of quality attributes of a biosimilar product is crucial to justify the future performance of narrowed clinical studies.

## 4. Conclusions

It was demonstrated that a biosimilar trastuzumab that exhibits comparable physicochemical and functional properties to the reference product also shows a comparable PK profile. Charge heterogeneity, glycosylation microheterogeneity, affinity constant to FcRn, and aggregates level were showed to be highly comparable between Trastuzumab-Probiomed and its reference product. In addition, the differences in the pharmacokinetic parameters between the two products were not statistically significant confirming that physicochemical and functional similarity of critical quality attributes resulted in similar pharmacokinetic profiles as expected. The information obtained here is valuable to be considered for upcoming comparability studies of biosimilars, since current state-of-the-art analytical methodologies allow the assessment of relevant quality attributes related to the safety and efficacy. Hence the necessity of conducting confirmatory extended nonclinical and clinical studies should be founded on the results coming from the physicochemical and biological characterization.

## Figures and Tables

**Table 1:** Isoelectric point by cIEF.

Brand	Batch	Main isoform (pI units)	Most acidic isoform (pI units)	Most basic isoform (pI units)	Global pI (pI units)
Herceptin	N35893	8.68 ± 0.01	7.93 ± 0.13	8.94 ± 0.03	8.60 ± 0.00
	B3423B020	8.70 ± 0.01	8.04 ± 0.10	9.00 ± 0.06	8.62 ± 0.01
	B3417B010	8.72 ± 0.01	8.04 ± 0.12	8.86 ± 0.03	8.62 ± 0.01

Trastuzumab-Probiomed	TZPP12002	8.69 ± 0.01	7.97 ± 0.11	8.97 ± 0.12	8.60 ± 0.01
	TZPP12003	8.69 ± 0.00	7.95 ± 0.08	8.84 ± 0.00	8.59 ± 0.01
	TZPP14001	8.69 ± 0.02	8.05 ± 0.14	8.99 ± 0.16	8.60 ± 0.03

Variation is presented as confidence interval at 95% (*n* = 3).

**Table 2:** Relevant glycoforms for PK by HILI-UPLC.

Brand	Batch	Hybrid (%)	High mannose (%)	Sialylated (%)
Herceptin	N35973	4.20 ± 0.18	4.40 ± 0.92	0.23 ± 0.09
	N35812	5.43 ± 0.22	3.14 ± 0.12	0.22 ± 0.03
	N36003	4.62 ± 0.18	4.34 ± 0.30	0.35 ± 0.11

Trastuzumab-Probiomed	TZPP12002	3.52 ± 0.11	2.27 ± 0.06	1.17 ± 0.15
	TZPP12003	3.64 ± 0.26	2.24 ± 0.17	1.29 ± 0.25
	TZPP14001	1.69 ± 0.07	1.50 ± 0.07	0.72 ± 0.02

Variation is presented as confidence interval at 95% (*n* = 3).

**Table 3:** Aggregates content obtained by SE-UPLC.

Brand	Batch	Aggregates (%)
Herceptin	N3597B013	1.06 ± 0.07
	N35973	0.21 ± 0.02
	B34310	0.22 ± 0.03

Trastuzumab-Probiomed	5423120902	0.39 ± 0.01
	5423120601	1.07 ± 0.03
	5422111201	0.25 ± 0.03

Variation is presented as confidence interval at 95% (*n* = 3).

**Table 4:** Pharmacokinetic parameters.

Brand	*C*_max_ (ng/mL)		AUC_0–*∞*_ (ng·h/mL)		MRT (h)	
	Mean	SD	Mean	SD	Mean	SD
Herceptin	74,428.3	28,504.1	11,746,396.0	4,377,022.8	174.9	49.2
Trastuzumab-Probiomed	73,353.1	11,463.8	13,611,268.7	5,053,533.5	153.9	51.2

SD: standard deviation.

**Table 5:** Mann-Whitney *U* test.

Parameter	*U* Mann-Whitney	*Z* score	*p* ^*∗*^
*C*_max_	71.00	0.381	0.7034
AUC_0–*∞*_	61.00	0.925	0.3551

^*∗*^If *α* ≥ 0.05  *H*_0_  (differences among values are not significant) is accepted; if *α* < 0.05  *H*_1_  (differences among values are significant) is accepted.
